# Population-Based Study of Epilepsy in Cambodia Associated Factors, Measures of Impact, Stigma, Quality of Life, Knowledge-Attitude-Practice, and Treatment Gap

**DOI:** 10.1371/journal.pone.0046296

**Published:** 2012-10-15

**Authors:** Devender Bhalla, Kimly Chea, Chamroeun Hun, Mey Vannareth, Pierre Huc, Samleng Chan, Robert Sebbag, Daniel Gérard, Michel Dumas, Sophal Oum, Michel Druet-Cabanac, Pierre-Marie Preux

**Affiliations:** 1 INSERM U1094, Tropical Neuroepidemiology, Limoges, France; University of Limoges, School of Medicine, Institute of Neuroepidemiology and Tropical Neurology, GEIST, Limoges, France; CHU Limoges, Limoges, France; 2 University of Health Sciences, Phnom Penh, Cambodia; 3 Department of Neurology, Calmette Hospital, Phnom Penh, Cambodia; 4 Department of Access to Medicines, Sanofi, Gentilly, France; 5 Cambodian Society of Neurology, Phnom Penh, Cambodia; University of Ottawa, Canada

## Abstract

**Purpose:**

Identify epilepsy-associated factors and calculate measures of impact, stigma, quality of life (QOL), knowledge-attitude-practice (KAP) and treatment gap in Prey Veng, Cambodia.

**Methods:**

This first Cambodian population-based case-control study had 96 epileptologist-confirmed epilepsy cases and 192 randomly selected matched healthy controls. Standard questionnaires, which have been used in similar settings, were used for collecting data on various parameters. Univariate and multivariate regression was done to determine odds ratios. Jacoby stigma, 31-item QOL, KAP etc were determined and so were the factors associated with them using STATA software. Treatment gap was measured using direct method.

**Key findings:**

Multivariate analyses yielded family history of epilepsy, difficult or long delivery, other problems beside seizures (mainly mental retardation, hyperthermia), and eventful pregnancy of the subject's mother as factors associated with epilepsy. There was high frequency of seizure precipitants esp. those related to sleep. Population attributable risk (%) was: family history (15.0), eventful pregnancy of subject's mother (14.5), long/difficult birth (6.5), and other problem beside seizures (20.0). Mean stigma (1.9±1.1, on a scale of 3) was mainly related to treatment efficacy. Mean QOL (5.0±1.4 on a scale of 10) was mainly related to treatment regularity. Cause or risk factor could be determined in 56% of cases. Treatment gap was 65.8%.

**Significance:**

Factors in pre- and perinatal period were found to be most crucial for epilepsy risk in Cambodia which inturn provides major prevention opportunities. A global action plan for treatment, stigma reduction and improvement of QOL should be set-up in this country.

## Introduction

Medical and social domains of epilepsy in Cambodia are poorly investigated. Based on a first-ever large population-based door-to-door representative study (N = 16510), the lifetime prevalence of epilepsy in Prey Veng province is 5.8/1000 [1] thus, on average, about 86000 people are estimated to have epilepsy (active and inactive) in Cambodia. Although this lifetime prevalence is different when compared to several individual Asian populations, it is not different from the median population-based lifetime prevalence in Asia (6.0/1000) [2]. Currently, factors associated with epilepsy in Cambodia are not known which are crucial for developing control or prevention strategies in this country. Coming to the social domain of epilepsy, it is evident that despite scientific advances stigma, misconceptions and negative attitude continue to exist and in turn impact the psychosocial health and quality of life (QOL) for people with epilepsy (PWE) in many populations [3]. However, it is likely that the nature of these impacts differ in Asia because of different socio-cultural environment than other resource-poor (REP) regions [4]. Thus we aimed to conduct a representative population-based study to firstly identify epilepsy-related factors in its entirety and secondly evaluate the stigma, quality of life, knowledge-attitude-practice (KAP), and treatment status associated with epilepsy in Prey Veng province, Cambodia.

## Methods (Study Area, Population, Data Collection, Data Analyses)

This population-based study was conducted in Prey Veng province and was an extension of the earlier population-based epilepsy prevalence study that was conducted in the same population [1]. In short, Prey Veng is densely populated and has about 1081609 people in 1142 villages. This region is fairly representative of the Cambodian demographic and environmental features [5]. This region was chosen because of its good accessibility from Phnom Penh. In short, 96 epileptologist-confirmed epilepsy cases were identified from the target population of all adults and children ≥1 year of age residing in the households of randomly selected villages (n = 24) within the Prey Veng Province. Two controls were randomly selected from the same source population and were matched for gender, age (±5 years in adults and ±2 years in children <15 years old), and village.

To determine case histories and KAP, we used detailed questionnaires that are already used in several population-based surveys in similar REP settings. Information on stigma was derived using the Jacoby stigma questionnaire [6] and information on QOL was collected using 31 item QOL in epilepsy (QOLIE-31) questionnaire [7]. A direct method was used to derive information on the treatment history by using a detailed questionnaire that has already been used in other surveys in REP settings. Standard epilepsy definition and seizure classification guidelines were used for cases [8]–[9]. For calculations, some results are reported in the descriptive manner (mean, median, standard deviation (SD)) such as demographic information, KAP, treatment etc. For calculations on the measures of association such as odds ratio, we used matched univariate and multivariate (backward stepwise) logistic regression analysis with two-sided 5% limit for statistical significance. Accepted formulations, for example Levin's formulation for population attributable risk, were used for deriving results of various measures of potential impact. Uncorrected chi-square was used. Data was analysed by using the STATA (version 8.0; STATA, Cary, NC) overall and for determining various predictors associated with stigma and QOL by performing logistic regression. Our case-control study had a power of nearly 80% (76.5% exactly) after assuming the exposure at 50% and alpha at 5% (Epi Info 6.0, Centers for Diseases Control, Atlanta, USA). Ethical clearance was obtained from the National Ethics Committee for Health Research, Ministry of Health of Cambodia which specifically approved this study. A verbal informed consent was obtained from each subject prior to their participation. In rural Cambodia, the majority of people are illiterate thus written consent may not be an appropriate practice and verbal explanation was anyhow necessary. Therefore, a consent form to obtain verbal consent from respondents was proposed and approved by the Ethics Committee together with the study protocol. Prior to the interview, our enumerator read carefully the consent form. This consent form contains information on the objectives of the study, the selection process, risks, benefits and freedom of the participation, as well as information on confidentiality. Only those who intended to give their consent were included as the study participant thus participation equated the documentation of their consent as well. None of the subject however had intended to refuse to participate.

## Results

### Study population

There were 96 cases and 192 matched controls. There were slightly more (53.1%) male epilepsy cases and the median age of the cases was 24.0 years, SD±13.6, range 3–70. Those <20 years represented 65.6% of the cases and the most frequent age group was 12–20 years (30.2%). On the other hand, 53.2% controls were males and the median age of controls was 28.0 years (SD±14.1, range 3–77). Five (2.6%) controls had some health problem, namely two subjects with chronic headache and one subject each with sleep disturbance, gastric ulcer, and weakness. All controls had a normal neurological clinical exam.

### Clinical description of cases

Generalised epilepsy consisted of tonic-clonic seizures (76.0%), absence (1.0%) and myoclonic seizures (3.1%) whereas partial epilepsy consisted of simple and complex partial seizures (12.5%), and olfactory partial seizures (1.0%). Mixed seizures consisted of 6.2% cases and 0.2% cases remained unclassified.

### Factors associated with epilepsy

Factors associated with epilepsy and their respective odd's ratios are provided in tables 1 and 2. Univariate analyses showed family history of epilepsy, uneventful pregnancy and use of drugs during pregnancy by subject's mother, long/difficult and premature birth of the subject, post-birth crying, current hospitalization for any cause, neurological sequel of other disease, having any other disorder beside seizures, mental retardation, history of head injury with loss of consciousness before seizure onset as factors linked to the risk of developing epilepsy. In the multivariate analyses, long or difficult birth of the subject, eventful pregnancy of the subject's mother, having any other disorder beside seizures and family history of epilepsy were found to be statistically significant for the risk of developing epilepsy. Further in a multivariate model after excluding “other problem beside seizures” (mental retardation and hyperthermia) as possible confounder, the odd's ratios that we obtained for remaining factors (i.e. long or difficult birth of the subject, eventful pregnancy of the subject's mother, and family history of epilepsy) were not different from the original multivariate result. These results are given in the table 2.

**Table 1:** Epilepsy associated factors in Prey Veng province, Cambodia (univariate analysis).

Parameter	OR crude	95% CI	p-value
Genetic factors			
Family history of epilepsy (any degree relative)	2.9	1.4–5.6	0.0018
Perinatal factors			
Uneventful pregnancy of the subject's mother	0.08	0.0–0.2	<0.0001
Long or difficult pregnancy	12.6	3.5–45.4	<0.0001
Premature birth	2.7	1.4–5.0	0.0013
Post-birth crying	0.3	0.1–0.9	0.03
Use of drugs during pregnancy	5.4	2.0–14.7	0.0009
Trauma/hospitalization related factors			
Currently hospitalised for any cause	4.3	1.7–10.5	0.0014
Head injury with loss of consciousness before seizure onset	7.4	2.3–23.5	0.0006
Comorbid factors			
Neurological sequel of any disorder	28.5	3.6–222.8	0.001
Beside seizure, any other disorders	8.2	2.9–23.1	<0.0001
Others			
Mental retardation	122.1	16.3–910.8	<0.0001

Footnote: CI: Confidence interval; OR: Odds ratio.

**Table 2:** Epilepsy associated factors in Prey Veng province, Cambodia (multivariate analysis).

Parameter	OR	95% CI	p-value
Family history of epilepsy	3	1.16–8.14	0.02
Eventful (hemorrhage, hypertension) pregnancy of the subject's mother	9.9	2.9–33.5	0.0002
Long or difficult birth	8.52	2.08–34.89	0.002
Other problems beside seizure**	13.66	2.68–69.48	0.001
After excluding other problems beside seizure (mental retardation, hyperthermia) from the model			
Family history of epilepsy	2.6	1.0–6.6	0.04
Eventful (hemorrhage, hypertension) pregnancy of the subject's mother	8.6	2.6–28.2	0.0004
Long or difficult birth	8.5	2.1–33.1	0.002

**Footnote:** CI: Confidence interval; OR: Odds ratio.

** Other problems beside seizure: mental retardation, hyperthermia.

### Seizure precipitating factors

We enquired about several seizure precipitants among our cases and their presence was positively reported in the following manner: having seizures within one hour of awakening from sleep (18.7%), lack of sleep (18.6%), during sleep (16.4%), stoppage of treatment (13.1%), emotional disturbance (12.0%), alcoholism (5.4%), menstruation (4.3%), and hyperventilation (3.1%). Rest did not report any seizure precipitant.

### Measures of impact

The difference in proportions of various risk factors between case and control population was calculated. Excess proportion of risk factors was observed among cases than controls in the following manner: abnormal pregnancy of the subject's mother (+22.3%, p<0.0001) followed by other problem beside seizures (+15.1%, p<0.0001), long or difficult birth (+14.1%, p<0.0001) and family history of epilepsy (+14.0%, p = 0.0006).

Case fraction (CF) is the proportion of exposed among total study population. These fractions were calculated for various risk factors in our population and were: 14.5% (95% CI 3–50) from positive epilepsy family history, 10.0% (95% CI 4–21) from eventful pregnancy of the subject's mother, 6.2% (95% CI 4.7–7.5) from long or difficult birth, and 7.6% (95% CI 5.9–8.2) from other problems beside seizures (mainly mental retardation and hyperthermia).

The Attributable risk indicates the amount of epilepsy that can be attributed to a factor among the group exposed to a risk factor. The fractions (in %) for various risk factors in our population were: 66.6% (95% CI 50–86) from positive epilepsy family history, 89.8% (95% CI 85–92) from eventful pregnancy of the subject's mother, 88.2% (95% CI 83–93) from long or difficult birth, and 92.6% (95% CI 88–95) from other problems beside seizures (mainly mental retardation and hyperthermia). These figures indicate that 66.6–92.6% of those exposed to one of these factors could avoid having epilepsy as outcome.

**Figure 1:**
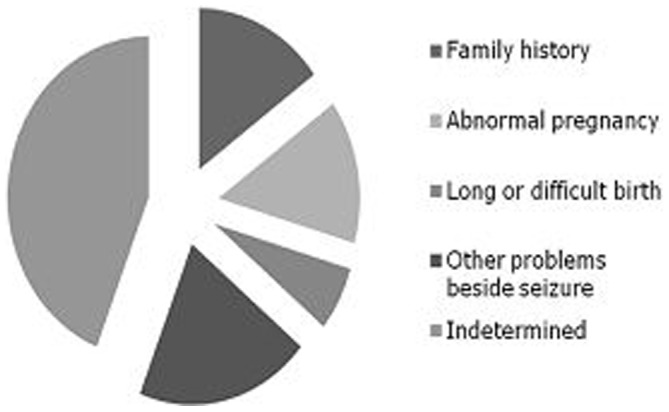
Causal pie model of epilepsy in Cambodia.

The population attributable risk percentage (PARP) expresses the increase of risk in the general population (over that for a population which had no exposure) which is due to the exposure. It is the measure of weight of risk factor on the occurrence of a disease and in turn indicates the proportion of cases that can be eliminated from the total population if the factor is eliminated. We calculated the attributable risk for each factor that we obtained by using standard formula for case-control studies. We obtained population attributable risk of 15.0% (95% CI 4–51) from positive epilepsy family history, 14.5% (95% CI 7–27) from eventful pregnancy of the subject's mother, 6.5% (95% CI 1–21.0) from long or difficult birth, and 20.0% (95% CI 9–41.0) from other problems beside seizures (mainly mental retardation and hyperthermia). This risk also implies that the relation of these factors with epilepsy is causal at least statistically and overall in Cambodia a cause/risk factor can therefore be evidently determined in at least 56.0% of cases. Causal Pie Model for population of Cambodia is given in Figure 1.

### Stigma

**Figure 2:**
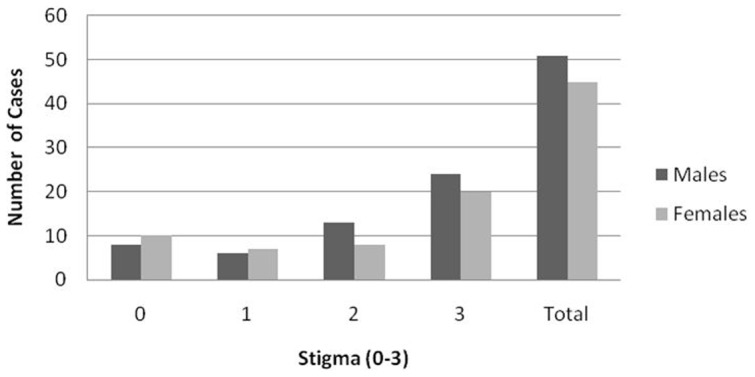
Gender- and degree-specific distribution of stigma related to epilepsy.

We enquired about stigma by using three-question jacoby scale (table 3, Figure 2). The mean stigma score in the overall population was 1.9±1.1 on a range of 0–3. Scores from none to highest stigma were reported in following number of cases: 18 (18.7%), 13 (13.5%), 21 (21.8%), and 44 (45.8%), figure 2. Mean stigma levels in males and females were 2.03 (SD±1.1) and 1.8 (SD±1.2) (p = 0.3). The factors significantly associated with stigma in our population were: *KAP* (considering epilepsy as curable p = 0.04, education of child is possible p = 0.02, marriage is possible despite having epilepsy p = 0.0001, ability to speak about epilepsy p = 0.003), *precipitants* (sleep disturbance p = 0.05), *seizure type* (tonic-clonic p = 0.05), *quality of life* (overall quality of life p = 0.002, quality of life in the last 4 weeks p = 0.0006, fear of subsequent seizures p = 0.06, limitation in memory due to epilepsy p = 0.0003, limitation in work due to epilepsy p = <0.000, social limitations due to epilepsy p = 0.06), *treatment* (treatment efficacy subject's view p = 0.03, treatment efficacy physician's view p = 0.02).

**Table 3:** Jacoby Stigma Scale.

Parameter	Not at all (%)	May be (%)	Certain (%)	No response (%)
Others being uncomfortable	26	45.8	27	1.2
Considered as inferior	31.2	48.8	18.7	1.3
Being avoided	43.7	41.6	13.5	1.2

### Knowledge-attitude-practice

Details are presented in table 4. *Knowledge:* In our population about half of cases (53.6%) considered epilepsy as contagious. Hereditary (41.5%), saliva (21.5%), sharing food plate (17.6%, sexual route (7.8%), rubbing shoulders or shaking hands (5.8%), gas emission (1.9%) and others (3.9%) were reported to be possible sources for contagion. Most (84.3%) cases considered their epilepsy as treatable and mainly by modern medicines (91.5%). Origin of epilepsy was largely unknown (56.3%) or natural (43.7%) and none of our cases reported origin of their epilepsy as supernatural. *Attitude:* Others were reported to be highly protective of cases (98.9%), education and marriage respectively was considered possible by 68.7% and 33.3% of cases. Limitation in marriage possibilities was considered to be due to hereditary nature of epilepsy (53.9%), fear of contamination (23.8%), inviting bad luck (15.8%) and fear of seizure (6.5%). Twenty-two (22%) cases reported taboos: fear of contamination (52.4%) and inviting bad luck (47.6%). *Practice:* Seizures were reported to interrupt work (63.5%) or to practice sports (30.3%) but they were reported to not interrupt travelling (47.9%), attending social functions (79.1%), talking with others about their epilepsy (57.2%), or sharing same smoke pipe and food plate (75%).

**Table 4:** Knowledge, attitudes and practices about epilepsy.

Parameter	Response (%)
Epilepsy is contagious	Yes 53.6; No 30.5; Unaware 15.9
Contagious by what	Hereditary 41.5; Saliva 21.5; Sharing food plate 17.6%; Sexual route 7.8; Rubbing shoulder/shaking hands 5.8; Gas emission 1.9%; Others 3.9%
Epilepsy is curable	84.3, non-curable 15.6
Curable by what	Traditional 2.4; modern 91.5; combination 6.1
Origin of epilepsy	Unaware 56.3; Natural 42.7; Supernatural 0,
Attitude of those around	Highly protective 98.9; No response 1.1
Education of epilepsy child is possible	68.7
Marriage of epilepsy child is possible	33.3
Why not	Hereditary 53.9; Fear of contamination 23.8; Avoid bad luck 15.8; Fear of seizure 6.5
Taboos exist	22
Examples of taboo	Fear of contamination 52.3; Avoid bad luck 47.6
Seizures interrupt work	63.5
Those around permit you to travel	47.9
Others DO NOT prevent you from using same smoke pipe or food plate	75
Seizure interrupt practicing sports	30.3
Others DO NOT prevent you from attending functions	79.1
Others allow you to talk about your disease	57.2

### Treatment status and treatment gap

All cases were enquired about their treatment history. Traditional treatment was being used by 18.7% (3.1% of cases had changed from drug therapy to traditional treatment) and was mainly prescribed by subject himself (40.0%) or by traditional healer (53.3%) or someone else (6.6%). Traditional treatment was mainly herbal (60%) and nature of treatment for rest was mineral, mixed or unknown. None had animal product based treatment. Only 4 cases (26.6%) were taking traditional treatment regularly and its efficacy (subject's view) was good (6.6%), moderate (13.3%), bad (46.6%) or indeterminable (33.3%). Modern treatment was being used by 34.3% cases (60.6% on modern monotherapy, 36.3% on modern plus traditional treatment, 3.0% changed from traditional to carbamazepine). Out of these, 42.2% were taking modern treatment regularly and its efficacy (subject's view) was good (21.2%), moderate (54.6%), bad (18.1%), or none and unknown in 6.0% cases. Forty-three percent (43%) were not on any treatment and treatment type was unknown in 4.1% cases. The treatment gap was 65.8%.

### Quality of life-31 (QOLIE-31)

**Figure 3:**
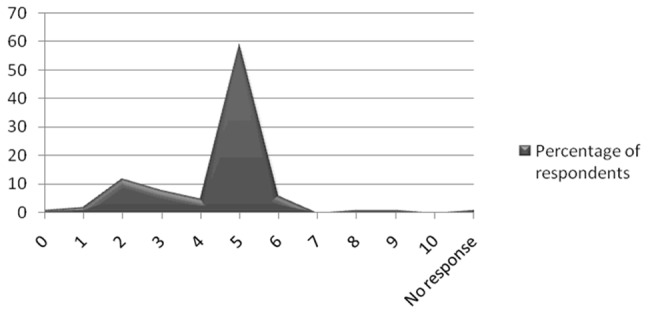
Respondents's self-reported quality of life (QOL) (0–10 indicates worst QOL to best QOL).

**Figure 4:**
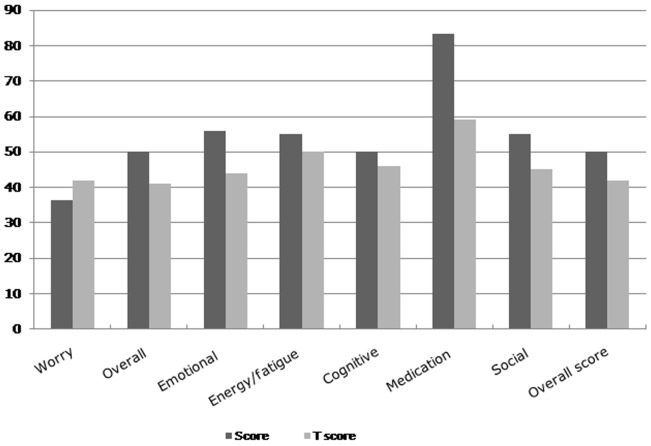
Scores of various quality of life subscales and their respective T-score.

The QOL using QOLIE-31 is measured on a scale from 0 to 10, with 10 representing the best possible quality of life and 0 representing worst possible quality of life. The median overall QOL was 5.0 (SD±1.4), figure 3. Median (and SD) scores for various QOL domains were: worry due to seizures (36.3±22.1), overall quality of life (50.0±15.2), emotional well-being (56.0±15.0), energy/fatigue (55.0±20.5), cognitive (50.2±15.4), medication effects (83.3±24.0), and social function (55.0±18.5). They are compared with relevant T-scores in the figure 4. On comparison with the T-scores, only worry score was lower than T-score and this difference was statistically significant (p = 0.01). Spearman's rho showed that QOL scores and t scores were statistically independent of each other (rho 0.75, p = 0.02). The factors that were found to be statistically associated with QOL were the following: *worry QOL* (regularity of treatment, coefficient −0.011, t −2.36, p = 0.02 and symptomatic epilepsy, coefficient −0.005, t−2.23, p = 0.02); *cognition QOL* (early age of onset, coefficient −0.007, t −2.13, p = 0.03). Factors that were considered to be nearly significant were the following: *cognition QOL* (tonic-clonic seizures, coefficient 0.004, t 1.70, p = 0.09), *medication QOL* (tonic-clonic seizure, coefficient −0.002, t −1.68, p = 0.09).

## Discussion

We conducted a first-ever large representative population-based matched case-control study in Cambodia that had epilepsy as a primary objective and used robust case-finding methods. All epilepsy cases were confirmed by epileptologists and each case had two matched controls randomly selected from the same source population. We aimed to identify factors that might have been associated with epilepsy in this population and in multivariate analysis, most of our risk factors were maternal-child-health related signifying the importance of this period for risk of having epilepsy. These factors are not different with results from other populations that had predominant generalised seizures (i.e. predominant absence of localised lesions) and epilepsy onset at a young age [10].

Factor that was most significantly (at least statistically) found was «other problems beside seizures» and this mainly included mental retardation and hyperthermia. Mental retardation is independently associated with the risk of having epilepsy [11]. Hyperthermia is likely an important factor for increased risk of having febrile convulsions which are an important risk factor for epilepsy [12]–[13] and exposure to maternal hyperthermia (likely a proxy of maternal infection) particularly increase the risk for epilepsy among their offsprings [12] [14]. This further signifies the importance of prenatal and perinatal period as a high-risk period related to the risk of having epilepsy. Hyperthermia also aggravates brain damage especially of the hippocampal area [15] and predisposes to learning difficulties manifested early or in the later life [16], in our study for example its relation with abnormal psychomotor development in childhood was statistically significant (p = 0.02). In another multivariate analysis, we excluded “other problem beside seizures” (mental retardation and hyperthermia) for possible confounding and the odds ratio that we obtained for remaining factors was not different from the odds ratios obtained from the original multivariate model. This implies that our pre- and perinatal factors are true risk factors for epilepsy, at least statistically (table 2).

Other factors that we obtained in our results were also pre- and perinatal such as eventful pregnancy of the subject's mother and long or difficult birth. These factors are independently and causally related to the development of lifetime and active epilepsy [17]–[18]. Results from developed [19] and resource-poor countries [20] confirm their association particularly antenatal bleeding, difficult labour. Our results therefore validate the idea of a «continuum of reproductive causality» [21] proposed in 1954 and indicate that epilepsy is initiated or exacerbated by prenatal events which makes this period as the most critical high-risk period for having epilepsy in Cambodia.

A positive family history increases the risk to have epilepsy by two- to three-fold [22], [23] excluding specific scenarios for instance among cases who develop epilepsy from other important factors such as trauma [24] or in patients with late onset [25]. We obtained a very similar 3-fold increase (p = 0.02) in the risk to develop epilepsy. Genetic influences are greater when the age of onset of epilepsy is young [26] and matches with the young median age of our population with 65.6% cases being <20 years of age.

Even though most seizures are considered to occur spontaneously, they can be precipitated by several endogenous or exogenous (seizure inducing and seizure triggering) factors. We enquired about presence of seizure precipitants and many cases reported a presence of seizure precipitants. Our frequency of precipitating factors is much higher than other studies [27] but similar factors were reported such as emotional stress, lack of sleep and tiredness. Stoppage of treatment as seizure precipitant was also important in our study. It might be important to assess their role in treatment outcome (such as in patients with low treatment responses) and their putative inclusion in regular epilepsy care [28].

Stigma is often associated with epilepsy [29] and can be present in at least 1/3^rd^ of epilepsy individuals [30]–[31]. In other Asian countries such as Laos or Vietnam, studies show high stigma [32]–[33]. In Europe where median epilepsy prevalence is similar to that observed in Asia, about 18% cases can be severely stigmatized [34], [35]. Mean stigma in our population was 1.9±1.1 on a range of 0–3 and about 46% cases reported highest stigma score, Figure 2 and table 3. Descriptively, there were more females who were free from stigma than males and there were more males who had highest stigma, Figure 2. Furthermore, stigma was statistically correlated with efficacy of the past modern treatment (both subject's, p = 0.03 and physician's evaluation, p = 0.02) and thus importance of seizure control for stigma reduction can be reemphasized here [34] [36]. Similar results have been observed where treatment reduced non-neurological stigma [37]. Many other factors were obtained that correlate with stigma in this population, for instance the seizure type (tonic-clonic, p = 0.05) and fear of subsequent seizure (nearly significant, p = 0.06), positive attitude about epilepsy for instance taking epilepsy as a curable disorder (p = 0.04) or having education (p = 0.02) and marriage (p = 0.0001) despite epilepsy [38], [39] or ability to speak about epilepsy (p = 0.003) [40]. Stigma is also correlated to quality of life in epilepsy cases [41]. In our population, having seizure precipitants particularly sleep disturbance (p = 0.05), limitations in memory (p = 0.0003), work (p<0.000) and social interactions (p = 0.06) were associated with stigma and so were the overall quality of life (p = 0.002), quality of life in last 4 weeks (p = 0.0006) in our population. Some of these factors are highlighted in other studies [42] and are undoubtedly important for positive social consequences [43] in this population also, some of these factors may likely act *vis-a-vis* thwarting concealment due to fear of stigma or of anxiety, shame or self-exclusion etc. [44] [45], [46].

Epilepsy since long is considered to be a contagious disorder and in our study nearly 54% cases reported epilepsy as a contagious disorder, mainly through hereditary route (41.5%), saliva (21.5%), sharing food plate (17.6%), sexual route (7.8%), rubbing shoulder/shaking hands (5.8%). However, some of the contagions reportedly observed in other populations were not reported by our population for instance reporting of breath, urine, faeces, blood, sperm and genital secretions as contagions [47]. Other important differences from other populations were absence of stigmatisation of whole family or village as reported in some African studies [48] and so was the absence of supernatural belief about epilepsy [49]. Also there were no difficulties related to sharing food, talking about epilepsy and attending social functions and most likely be due to the close social ties and open housing in this rural population that facilitate positive social support and interaction on a daily basis and protection for others. Almost all cases (98.9%) had reported that others are protective of them. Also despite rural nature of the population and limited knowledge about epilepsy majority of our cases considered epilepsy as a curable disorder and by modern treatment. This also reflect in the fact that only minority (about 19%) had exclusively sought traditional treatment.

Median overall QOL in our population was 5.0±1.4, Figure 3. Except for seizure worry, scores for other domains were at least 50%. This is slightly better than other nearby Asian populations [50] or Russia [51] which have reported lower overall mean score. QOL scores in our population were not better than resource-rich populations [52] which might be due to better healthcare possibilities or with respect to different sociocultural environments [53].

The score for seizure worry was (36.3±22.1) lowest and highest for medication effects (median score of 83.3±24.0), a pattern that is also seen in nearby Asian populations [50] but different from some European populations [54]. Highest subscale score for medication effect could partially reflect the absence of influence from the treatment-related side effects since large proportion of cases were not on any treatment thus possible side effects from treatment may have not affected their daily lives. Treatment regularity was statistically associated with low seizure worry (p = 0.02) in our population an effect that is expected and observed in other studies as well [55]. Having symptomatic epilepsy was also statistically correlated with increased worry for seizures (p = 0.02), a seizure severity might be a possible explanation. Type of seizure (tonic-clonic seizure, p = 0.09) was related to the cognitive and medication effect subscales (p = 0.09) although only at a 9% statistical significance. This observation nonetheless matches with results from other studies [56]. Patients with epilepsy often experience cognitive dysfunction either directly as an effect of epilepsy or due to its treatment [57]. Early age of onset (<5 years age) was correlated with the cognitive subscale (p = 0.03) in our population and is in concurrence with the results from many other studies that report increased subsequent vulnerability of cognitive impairment, in both mature and immature brain, due to early-age onset. Other studies indicate that this effect manifest early in the course of epilepsy and might manifest through higher risk of mental retardation associated with early onset in epilepsy children [58] or as harmful effect of anti-epilepsy treatment itself [59].

Treatment gap that is observed in our population is not different from some populations although much lower than some other populations such as Pakistan, Laos etc [2]. It is not unknown that the treatment opportunities are limited in most resource-limited countries for multitude of reasons. The difference from some other populations was the frequency and the nature of the traditional treatment in our population possibly due to the lack of efficacy (79.9% considered treatment effect bad or indeterminable) of this treatment in our population. This type of treatment is predominantly sought in many other populations [60] possibly may lead to hypothesize about the difference in type and efficacy of traditional treatments of Asia and other regions such as sub-Saharan Africa. Other Asian study also reported traditional epilepsy treatment may not necessarily be the primary mode of treatment [61]. Modern treatment was being used by about 34% cases and its effect was good to moderate in 75.8% cases, which may correlate with general observation that currently available treatments are sufficiently effective in many cases. However about 42% were taking this treatment regularly and again likely due to multitude of reasons observed elsewhere as well [2]. In case of Cambodia, National survey about access to healthcare showed that no one to accompany is one of the major hindrance along with cost and distance etc [5]. It is also likely that the knowledge of the health staff, low local production of medicines etc may particularly contribute towards treatment gap as observed in an unpublished study in Laos.

Strengths of this study lies in its population-based design and use of standard procedures for epilepsy cases and their controls. Results from all major aspects of epilepsy are addressed in this study. Weakness of our study is similar to other case-control studies conducted in such settings due to the possibility of recall bias.

## Perspectives

This study identified challenges and opportunities for Cambodia in relation to epilepsy, table 5.

**Table 5:** Opportunities and challenges for Cambodia.

*Challenges for Cambodia*
Lack of treatment opportunities by modern anti epilepsy treatment
Frequent seizure-precipitating factors
Poor QOL possibly related to various precipitating factors and lack of treatment opportunities or absence of alternative compounds or severity of epilepsy itself
Moderately high level of stigma. 46.0% cases with highest stigma particularly males
Epilepsy is a contagious disorder for about 54.0% cases (mainly hereditarily)
Origin of epilepsy is unknown for 56.0% cases
Can't freely practice sports or work
*Opportunities for Cambodia*
Major prevention opportunities exist. Strengthening of usual maternal-child health service can reduce number of new epilepsy cases by at least 41.0%
Epilepsy is a treatable disorder for 84.0% cases
Epilepsy is considered to be treatable by modern medicines rather than traditional
Epilepsy is not a supernatural disorder for all cases
Stigmatization of whole family and village is inexistent
Extensive social support (others are protective) for 99.0% cases
No limitations for education in 69.0% cases
Can freely share smoke pipe and food plate or attend social functions
More females than males are absent of any stigma

### Prevention

Our results show that in at least 56.0% of cases the causative or risk factor can be evidently determined in Cambodia. The epilepsy-associated factors (pregnancy events, long and/or difficult delivery, mental retardation, febrile seizures) obtained in this population are preventable in nature. These factors thus provide opportunity for prevention by at least 41.0% should exposure to these risk factors is eliminated in the general population of Cambodia. Prevention of epilepsy can be aimed through strengthening of usual maternal and child health service (MCH). This prevention proportion matches with the results from other resource-poor populations through control of similar factors [10]. Further studies should test the strategies and corroborate these results in this population.

### Treatment

Consistent and sufficient treatment remains one major challenge particularly in many resource-poor populations. Since traditional healers are usually approached for treatment needs, by minority of cases in this population though, it might be necessary to engage them in case identification, ascertainment and their treatment in future studies. They might be essential to bridge the gap between treatment and the target population since it is likely that neither traditional nor modern treatment can sufficiently address all epilepsy individuals alone.
